# TOR Signaling as a Central Integrator of Embryogenic Reprogramming During 2,4-D-Induced Somatic Embryogenesis

**DOI:** 10.3390/ijms27146191

**Published:** 2026-07-10

**Authors:** José Luis Cabrera-Ponce, Alex Ricardo Bermudez-Valle, Maria del Rosario Cárdenas-Aquino, Andrea Maria Navarro-Vega, Braulio Uribe-Lopez, Aaron Barraza-Celis, Eliana Valencia-Lozano, Lisset Herrera-Isidron

**Affiliations:** 1 Departamento de Ingeniería Genética, PlanTECC, Centro de Investigación y de Estudios Avanzados del IPN, Unidad Irapuato, Irapuato 36824, Guanajuato, Mexico; jlcabre@yahoo.com.mx; 2 Departamento de Biotecnologia y Bioquimica, Centro de Investigación y de Estudios Avanzados del IPN, Unidad Irapuato, Irapuato 36824, Guanajuato, Mexico; alex.bermude@cinvestav.mx (A.R.B.-V.); braulio.uribe@cinvestav.mx (B.U.-L.); 3 Departamento de Ingeniería Genética, Centro de Investigación y de Estudios Avanzados del IPN, Unidad Irapuato, Irapuato 36824, Guanajuato, Mexico; mrca85@ciencias.unam.mx; 4 Unidad Profesional Interdisciplinaria de Ingeniería Campus Guanajuato (UPIIG), Instituto Politécnico Nacional, Av. Mineral de Valenciana 200, Puerto Interior, Silao de la Victoria 36275, Guanajuato, Mexico; anavarrov1900@alumno.ipn.mx; 5 Laboratorio Biotechnolgika, Ignacio Ramirez 3765, La Paz 23060, Baja California Sur, Mexico; abarrazc@gmail.com; 6 Laboratorio de Investigación Interdisciplinaria (LII), Universidad Nacional Autónoma de México, Escuela Nacional de Estudios Superiores, Unidad León, León de los Aldama 37684, Guanajuato, Mexico

**Keywords:** *Arabidopsis thaliana*, somatic embryogenesis, TOR signaling, molecular mechanism, master regulators of somatic embryogenesis, ribosomal protein, DNA repair, carbon metabolism

## Abstract

2,4-Dichlorophenoxyacetic acid (2,4-D), originally developed as a synthetic auxinic herbicide, is the most widely used chemical inducer of somatic embryogenesis (SE) in plants. Despite extensive use of 2,4-D in plant regeneration, the systems-level regulatory mechanisms connecting hormonal signaling, metabolic reprogramming, translational control, and embryogenic competence remain poorly resolved. Here, we hypothesize that TOR signaling functions as an integrative molecular hub coordinating transcriptional, metabolic, and developmental reprogramming during somatic embryogenesis induction. To investigate the molecular regulatory landscape associated with 2,4-D-induced SE, we performed a systems-level analysis integrating publicly available transcriptomic data from *Arabidopsis thaliana* with high-confidence protein–protein interaction (PPI) network analyses using STRING v12.0 (confidence score ≥ 0.900). Using a previously published transcriptomic dataset, we identified 1927 upregulated genes associated with SE induction, which were organized into 34 functional modules related to transcriptional regulation, translation metabolism, hormone signaling and cellular homeostasis. Within this interactome, TARGET OF RAPAMYCIN (TOR) kinase emerged as an integrative regulatory hub associated with multiple pathways involved in embryogenic reprogramming. Network analyses revealed three major TOR-associated regulatory axes: (1) the *TOR–FKBP12–RPS6A* axis, associated with ribosome biogenesis and translational regulation; (2) the *TOR–CBP20* axis, connected with transcriptional reprogramming; SE master regulators (*LEC1*, *LEC2*, and *FUS3)*; and lipid, sterol, brassinosteroid (BR), and auxin-associated pathways; and (3) the *TOR–TAP46* axis, linked with one-carbon metabolism, nucleotide biosynthesis, DNA replication and repair, and genome-stability pathways. Additionally, the network contained 411 embryo-lethal (EMBL) genes distributed across multiple regulatory modules, reinforcing the biological relevance of the identified interactome and highlighting the importance of coordinated developmental, metabolic, and transcriptional regulation during embryogenesis induction. These findings support a systems-level TOR-associated regulatory framework involved in the integration of transcriptional, translational, metabolic, hormonal, and genome-maintenance pathways during embryogenesis. This interactome model provides a foundation for functional studies aimed at dissecting the molecular mechanisms underlying SE and identifying candidate targets to improve regeneration and biotechnological application and crop genetic engineering. Collectively, this study proposes a mechanistic framework in which TOR signaling integrates developmental, metabolic, translational, and genome-stability pathways to orchestrate embryogenic competence, providing candidate molecular targets for improving plant regeneration and genome engineering platforms.

## 1. Introduction

### 1.1. Auxins and the Emergence of 2,4-D

Auxins are central phytohormones that regulate a wide array of developmental and adaptive processes in plants, including organogenesis, cell elongation, and branching, as well as responses to environmental stimuli such as gravity, light, and mechanical signals [1]. The principal natural auxin, indole-3-acetic acid (IAA), was first identified in the 1930s and became a key compound in understanding plant growth regulation.

At that time, it was discovered that IAA could inhibit the growth of and kill certain broad-leaved weeds in cereal when applied at high concentrations (1 g/L) [2,3,4], without harming the grain crops [4]. However, IAA was too expensive and was too quickly degraded by microorganisms in the soil to be of practical agricultural use.

The complex chemical structure of IAA was not a barrier, since four independent research groups [4,5] were able to synthesize an array of IAA derivatives, including 1-naphthalene acetic acid (1-NAA) and the phenoxycarboxylic acids 2-methyl-4-chlorophenoxyacetic acid (MCPA) and 2,4-dichlorophenoxy acetic acid (2,4-D), which were among the most auxin-active molecules tested [6,7,8]. In 1945, 2,4-D under the names Weedone in the USA and Methoxone in the UK, began to be marketed as an herbicide.

Beyond its herbicidal use, 2,4-D has gained prominence as a powerful chemical inducer of somatic embryogenesis (SE)—a process in which somatic cells are reprogrammed to form embryos that can develop into whole plants.

Unlike zygotic embryogenesis, which occurs naturally via fertilization and includes the formation of the embryonic sac, endosperm, and seed coat, SE bypasses these structures. Instead, it can be artificially induced under in vitro conditions and provides a genetically identical route to clonal propagation.

Although the auxinic activity of 2,4-D has been extensively characterized, the molecular mechanism by which this synthetic auxin triggers large-scale cellular reprogramming toward embryogenic competence remains insufficiently understood.

### 1.2. Biological Basis of Somatic Embryogenesis

The SE process has become a valuable tool in plant biotechnology, with applications in synthetic seed production, cryopreservation, genetic transformation, and genome editing. The process involves the reprogramming of somatic cells into embryogenic stem cells, followed by differentiation into embryo-like structures. SE therefore represents an exceptional model for investigating how differentiated somatic cells reacquire totipotency through coordinated transcriptional, epigenetic, metabolic, and hormonal reprogramming.

This cellular transition requires the precise transcriptional regulation of numerous genes in response to stress signals, including hormonal imbalances, osmotic stress, salinity, heavy metals, and other signaling molecules. Accumulating evidence suggests that embryogenic induction requires extensive remodeling of cellular homeostasis, including chromatin accessibility, translational capacity, energy metabolism, and stress-response pathways.

### 1.3. Molecular Regulation of Embryogenic Competence

Transcriptomic studies across multiple plant species have identified large sets of differentially expressed genes (DEGs) during SE [9,10,11,12,13,14,15,16,17]. In *Arabidopsis thaliana*, a pivotal study by Wickramasuriya and Dunwell [18] (2015) provided a comprehensive transcriptomic dataset capturing gene expression patterns during 2,4-D-induced SE at 5, 10, and 15 days of induction.

From these datasets, DEGs with average log2 fold-change values greater than 4 were selected to construct a high-confidence (0.900) protein–protein interaction (PPI) network using the STRING v12.0 [19] database, and the network was visualized with Cytoscape 3.10.4.

**Figure 1:**
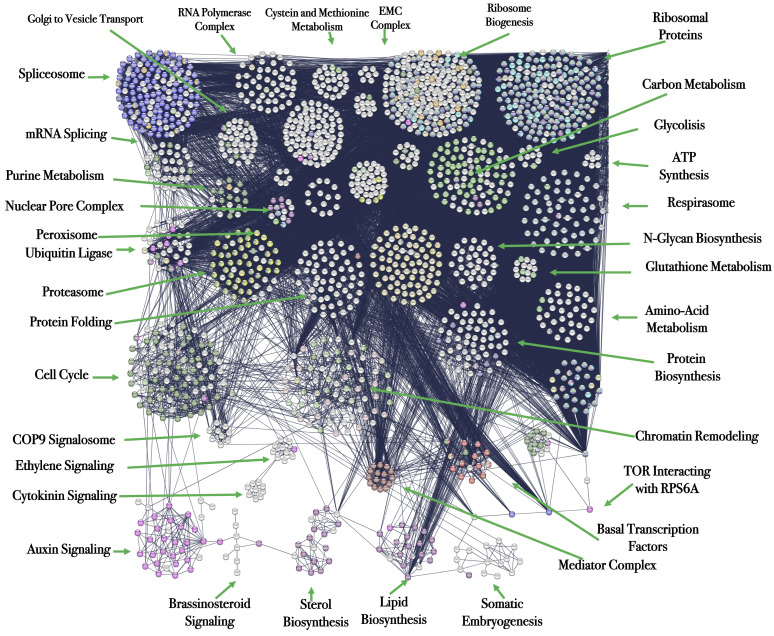
PPI network of 1927 upregulated genes involved in SE development of *A. thaliana*. The PPI is composed of 34 modules and was constructed using the highest confidence score (0.900).

Among the 16,034 genes upregulated during SE, 1927 were found to be tightly connected within the network, forming 34 distinct functional modules (Figure 1), and an analysis comparing network topology across multiple STRING confidence thresholds has been incorporated in Supplementary Table S4. These pathways are related to hormone signaling, transcriptional regulation, metabolism, proteostasis, and embryogenic development. However, most previous studies have focused on isolated regulatory components, whereas the systems-level coordination among transcriptional regulators, metabolic pathways, translational machinery, and signaling hubs during SE remains largely unresolved.

In this review, we integrate transcriptomic and interactomic analyses to reconstruct the systems-level regulatory architecture underlying 2,4-D-induced SE, with a special emphasis on TOR-centered signaling networks potentially coordinating embryogenic reprogramming. These will provide a systems-level understanding of SE and identify potential targets for experimental validation.

### 1.4. Systems-Level Transcriptomic Reconstruction of Embryogenic Regulatory Networks

The understanding of SE at the systems level requires the integration of high-resolution transcriptomic data with functional interaction networks. In *A. thaliana*, Wickramasuriya and Dunwell [18] (2015) provided a foundational dataset capturing gene expression during SE induced by 2 mg/L of 2,4-dichlorophenoxyacetic acid (2,4-D) under controlled photoperiod and temperature conditions.

The tissues were harvested at 5, 10 and 15 days post-induction, and the average gene expression values across these time points were determined.

To identify the most robust transcriptional responses associated with somatic embryogenesis induction, genes exhibiting average log2 fold-change values greater than 4 across the analyzed induction stages (5, 10, and 15 days) were selected for network reconstruction. This stringent threshold was intentionally applied to prioritize genes showing sustained and strong transcriptional activation during embryogenic reprogramming while minimizing the inclusion of weakly induced or potentially transient responses. The resulting dataset contained 1927 highly upregulated genes that were subsequently used for protein–protein interaction network analysis.

Because the original transcriptomic dataset was obtained from a previously published study, differential expression statistics were generated by the original authors. Consequently, gene selection in the present study was based on reported expression fold-change values rather than re-analysis of raw transcriptomic data (Supplementary Table S1).

### 1.5. Central Hypothesis

We propose that TOR signaling functions as a central integrative hub linking transcriptional activation, ribosome biogenesis, metabolic reprogramming, hormonal signaling, and genome-maintenance pathways during 2,4-D-induced somatic embryogenesis. Under this framework, embryogenic competence emerges from coordinated multi-layered regulatory interactions rather than isolated signaling modules.

### 1.6. Objective and Scope

**Figure 2:**
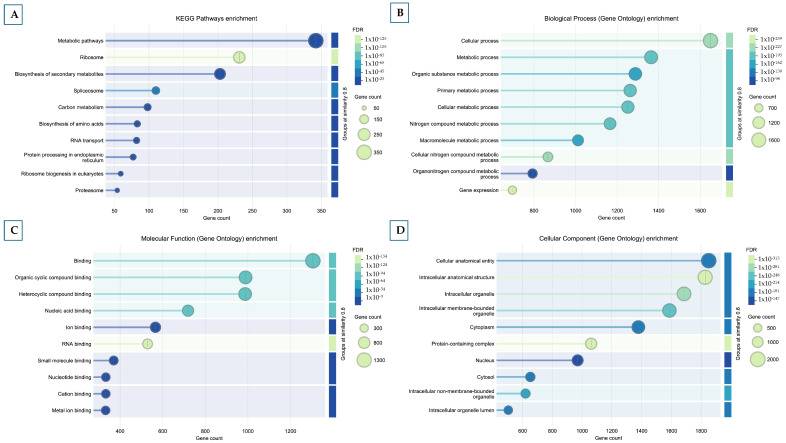
GO and KEEG enrichment analysis of 1927 upregulated genes involved in SE development of *A. thaliana*.

To contextualize the functional architecture of these genes, we categorized them into 34 functionally coherent modules (Figure 1), and GO biological, cellular, molecular and KEEG enrichment analysis was performed (Figure 2).

These modules are essential cellular processes and developmental pathways that are dynamically regulated during the progression of SE. The main functional groups included:

→ Transcriptional regulation: basal transcription factors, Mediator complex, and RNA polymerase machinery.
→ Translation and ribosome biogenesis: ribosomal proteins, ribosome assembly, and translation initiation factors.
→ Protein homeostasis: ubiquitin–proteasome system and COP9 signalosome.
→ Chromatin remodeling and nucleosome assembly.
→ Cell cycle and genome maintenance: DNA replication and repair.
→ Metabolism and biosynthesis: sterols, lipids, amino acids, nucleotides, and one-carbon metabolism.
→ Organelle and energy-related modules: mitochondrial protein import, ATP synthesis, and respirasome.
→ Hormonal signaling: auxins, cytokinins, ethylene and BRs.

This review aims to establish a mechanistic systems-biology framework explaining how TOR-associated regulatory modules may coordinate the acquisition of embryogenic competence during SE induction.

Particular emphasis is placed on transcriptional regulation, lipid and sterol metabolism, hormone signaling integration, translational regulation, and genome-stability pathways.

Methodological framework

Among these, a distinct cluster enriched in SE-associated genes was identified, suggesting the presence of a specialized gene set tightly associated with embryogenic reprogramming.

Furthermore, the network contained 411 embryo-lethal (EMBL) genes, whose loss-of-function phenotypes result in early embryonic arrest or failure. These EMBL genes were distributed across multiple modules, reinforcing the idea that successful SE requires the precise orchestration of diverse biological processes.

The presence of EMBL genes across transcriptional, translational, metabolic, and hormonal modules further supports the biological relevance of the identified network architecture. Their distribution indicates that many components within the network are essential not only for normal embryonic development but also for in vitro somatic reprogramming.

This integrated transcriptomic–interactomic framework reveals the complexity and coordination potentially required for SE and provides a functional roadmap for identifying candidate regulatory nodes, including TOR kinase.

Within this framework, TOR was identified as a putative integrative signaling node linking modules associated with transcription, translation, metabolism, and cellular homeostasis.

These observations support the hypothesis that TOR-associated regulatory networks may contribute to coordination of transcriptional, translational, metabolic, and developmental programs required for SE induction.

In the following section, we examine the role of TOR as a regulator of SE and its integration into the broader signaling landscape induced by 2,4-D.

Transcriptomic filtering criteria:

Only genes exhibiting average log2 fold-change values >4 across the three induction stages were retained. This conservative threshold was selected to focus the network analysis on genes displaying strong and persistent activation during somatic embryogenesis induction and to reduce the influence of low-amplitude transcriptional responses.

STRING rationale:

A stringent STRING confidence threshold (≥0.900) was selected to reduce false-positive interactions and identify highly reliable regulatory modules.

Network significance:

The resulting network architecture enabled identification of densely interconnected functional modules potentially associated with embryogenic competence acquisition.

Relevant Sections

## 2. The TOR Kinase as a Central Hub in SE

### 2.1. Overview of TOR Signaling in Plants

The TOR enzyme is an evolutionarily conserved kinase that plays a central role in regulating growth, metabolism, and lifespan in *A. thaliana*. TOR coordinates key cellular processes such as DNA replication, transcription, RNA synthesis, ribosome biogenesis, the biosynthesis of proteins, amino acids, lipids, nucleotides, and cell wall components involved in signaling in response to nutrient and energy availability [20].

TOR also supports metabolic and redox homeostasis by coordinating glycolysis, the tricarboxylic acid (TCA) cycle, glutathione biosynthesis, and Root Growth Factor (RGF) signaling [21].

Mutations in TOR lead to early embryo-lethal (EMBL) phenotypes, highlighting its essential role during embryogenesis and post-embryonic development [22,23]. Mutant embryos display reduced rRNA levels, suggesting that proper ribosome biogenesis is required beyond the 32-cell stage to maintain protein synthesis and other TOR-mediated functions [24].

TOR also modulates translation by phosphorylating ribosomal protein S6 (RPS6A) and activating S6 kinase (S6K1), which promote selective translation re-initiation of mRNAs associated with stem cell maintenance and differentiation [25,26,27].

In addition, TOR contributes to chromatin remodeling. Non-phosphorylated RPS6 binds to Histone Deacetylase 2B (HD2B) to suppress rDNA transcription, whereas its phosphorylation, potentially mediated through TOR signaling, releases this inhibition and promotes rDNA expression [28,29].

TOR signaling may therefore contribute to the coordination of molecular pathways underlying embryogenic competence and osmotic reprogramming.

### 2.2. Three Regulatory Axes of TOR Signaling During SE

Based on a PPI network comprising 1927 genes involved in SE, we identified putative TOR-associated interaction modules and functionally connected to TOR signaling during embryogenic reprogramming.

**Figure 3:**
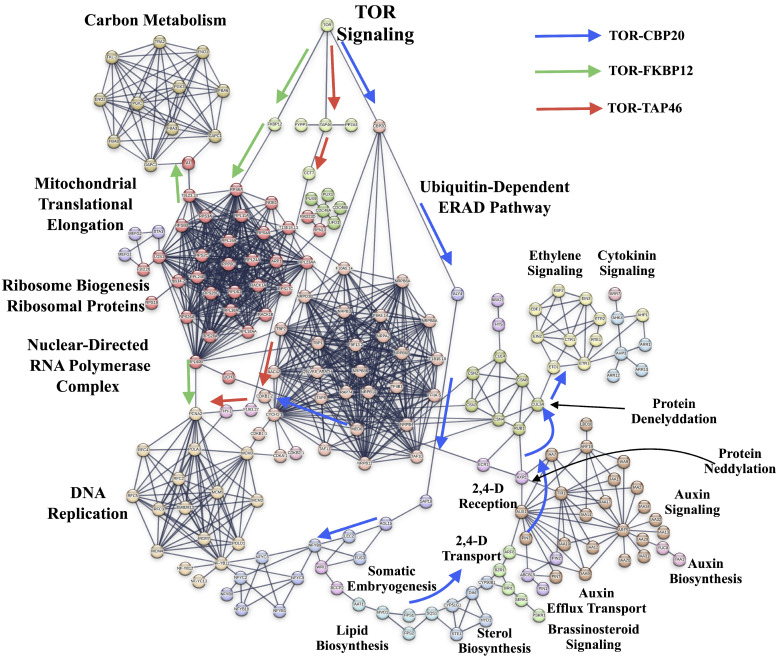
In the SE PPI network of *A. thaliana*, TOR interacts with FKBP12 (green arrows), CBP20 (blue arrows), and TAP46. FKBP12 is associated with RPS6A activation and linked to ribosome biogenesis and carbon metabolism modules; CBP20 interacts with SE-related transcriptional regulators and lipid, sterol, BR, and auxin biosynthesis pathways; TAP46 is associated with transcriptional regulation, one-carbon metabolism, and cell cycle/DNA repair modules. All three regulatory branches converge on genome-stability- and DNA repair-associated pathways. The network was generated using STRING v12.0, with a confidence score ≥ 0.900.

This analysis revealed 190 high-confidence TOR-associated interactions, which clustered into three major regulatory axes mediated through interactions involving FKBP12, CBP20, and TAP46 (Figure 3).

These modules were associated with translational regulation, transcriptional control, metabolic reprogramming, and genome maintenance during SE. The list and functional annotation of TOR-associated genes identified during SE are provided in Supplementary Table S2.

## 3. Discussion

### 3.1. TOR-CBP20 Interactions with Basal Transcription Factors and the Mediator Complex

**Figure 4:**
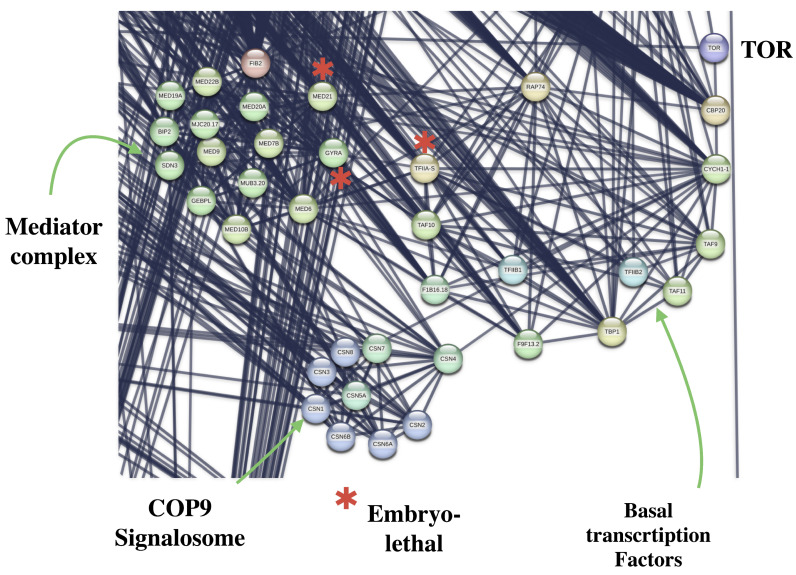
TOR–CBP20-associated interaction with basal transcription factor CYCH1-1, the Mediator complex, and COP9 signalosome. The PPI network was generated using STRING v12.0 with the highest confidence score (≥0.900).

TOR interacts with CBP20, which in turn is connected to the basal transcription factor CYCH1-1 (Cyclin-H1-1) within the SE-associated PPI network. CYCH1-1 is linked to CDK-2 and CDK-3, components potentially involved in the activation of cyclin-dependent kinase complexes associated with transcriptional regulation and cycle progression (Figure 4).

These interactions suggest a potential association between TOR signaling and the transcriptional machinery required for embryogenic reprogramming.

Basal transcription factor genes are essential for plant viability, as mutations affecting these components frequently result in EMBL phenotypes due to defects in cell growth, development, and transcriptional regulation during embryogenesis. The transcription initiation factor IIA subunit 2 (TFIIAγ) is indispensable for basal transcription in plants, and its loss leads to EMBL phenotypes. Within the SE-associated PPI network, TFIIA-S interacted with 33 proteins related to basal transcription factors; the Mediator complex; and RNA polymerase I, II and III activities in the functional enrichment analysis of TOR-associated regulatory axes, as described in Figures S1 and S2.

CYCH1-1 was also associated with MED6 (Mediator of RNA polymerase II transcription subunit 6), a component of the Mediator complex that acts as a coactivator involved in the regulated transcription of RNA polymerase II-dependent genes.

The Mediator functions as a molecular bridge transmitting signals from gene-specific regulatory proteins to the basal RNA polymerase II transcription machinery.

Upon recruitment of promoters through interactions with transcriptional regulators, the Mediator complex facilitates the assembly and stabilization of the preinitiation complex (PIC) together with RNA polymerase II.

Beyond its role in basal transcription, the Mediator complex integrates developmental programs, phytohormone signaling, and environmental stress responses [30].

Transcription factors recruit Mediator through interactions with tail subunits, which subsequently engage the RNA polymerase II C-terminal domain (CTD), thereby contributing to PIC stabilization [31].

*MED21* and *GYRA* have been identified as EMBL-associated genes within this regulatory framework. *MED21*, a middle-module Mediator subunit, is required for embryonic viability, and homozygous loss-of-function mutations result in EMBL phenotypes [32].

MED21 also interacts with the transcriptional corepressor TOPLESS (TPL), contributing to transcriptional repression pathways [33]. Likewise, GYRA, a type II topoisomerase localized in plastids and mitochondria, is required for the maintenance of DNA topology, and loss-of-function of *gyrA* mutants exhibit EMBL phenotypes [34].

These observations suggest that TOR signaling may participate in large-scale transcriptional reprogramming required for the transition from somatic identity toward embryogenic competence.

The association between TOR, Mediator components, and RNA polymerase-associated pathways further supports a role for TOR in coordinating global transcriptional activation during developmental reprogramming.

### 3.2. CYCH1-1 Basal Transcription Factor Interacts with COP9 Signalosome

CYCH1-1 was also associated with CSN4 (COP9 Signalosome complex subunit 4), a component of the COP9 signalosome complex (CSN). The CSN is a conserved regulator of the ubiquitin–proteosome system (UPS), which controls protein through ubiquitination and subsequent degradation.

The UPS is essential for embryogenesis because the precise timing, interaction, and turnover of regulatory proteins are required for proper cell cycle progression, developmental transition, and cellular differentiation during early embryo formation.

Ubiquitination contributes to these processes by promoting the selective degradation or inactivation of key regulatory proteins, thereby maintaining protein homeostasis and enabling coordinated responses to developmental and environmental signals.

At the molecular level, the CSN mediates deneddylation of the cullin subunits of SCF-type E3 ligase complexes, thereby modulating SCF ubiquitin ligase activity. Both neddylation and deneddylation are required for the proper assembly and activity of ubiquitin ligases and ultimately influence substrate degradation processes [35].

Loss of *CSN4* function results in severe developmental abnormalities and EMBL phenotypes, highlighting its essential role in plant growth and embryogenesis. Furthermore, double knockdown of both *CSN4* and *TCTP* causes plant lethality, indicating that complete disruption of CSN4-associated pathways is incompatible with survival [36].

CYCH1-1 was also associated with CSN3. Plants carrying disrupted *csn3* alleles die during the seedling stage, suggesting that normal post-germination development is dependent on proper CSN function.

Further downstream, CSN3 and CSN4 interacted with CUL1, a core component of SCF ubiquitin ligase complex. Loss of functional *CUL1* disrupts SCF-mediated ubiquitination, impairing cell division and developmental progression, ultimately resulting in EMBL phenotypes.

CUL1 also interacts with TIR1, a central auxin receptor involved in auxin-dependent transcriptional regulation. Although *tir1* single mutants are viable, complete disruption of the TIR1/AFB auxin receptor family results in severe embryonic defects and seed abortion, reflecting the essential role of auxin signaling during embryogenesis.

These observations suggest that TOR-associated transcriptional modules may be functionally connected with UPS-dependent regulatory pathways required for protein turnover, auxin signaling, and developmental progression during SE.

The integration of CSN- and SCF-associated components within the SE network further supports the importance of controlled proteostasis during embryogenic reprogramming.

The convergence of TOR-associated pathways with ubiquitin-mediated proteostasis suggests that embryogenic induction requires dynamic turnover of regulatory proteins to sustain developmental plasticity.

### 3.3. CBP20 Interactions with the Master Regulators of SE

TOR is a serine/threonine protein kinase that functions as a master regulator of cell growth and developmental progression from embryogenesis to seed production.

By integrating nutrient and energy availability, TOR influences key biosynthetic pathways, including those associated with cell wall biosynthesis and cellular proliferation.

**Figure 5:**
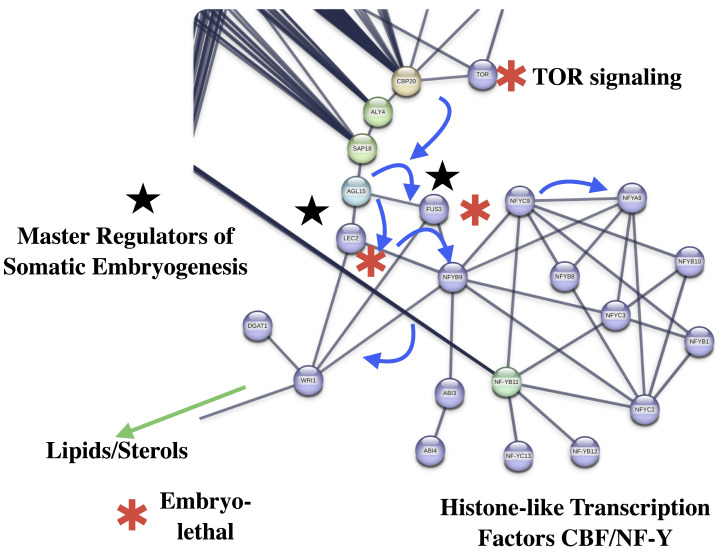
TOR interaction with CBP20 and master regulators of SE. The PPI network was generated using STRING v12.0 with the highest confidence score (≥0.900).

TOR interacts with CBP20 (Nuclear cap-binding protein subunit 2), a component of the cap-binding complex (CBC) (Figure 5).

The CBC participates in miRNA-mediated RNA interference and is required for primary miRNA processing. *cbp20* mutants exhibit altered expression of *LEC1*, *LEC2* and *FUS3,* resulting in impaired SE, altered abscisic acid signaling and reduced embryogenic competence [37]. In addition, the *acbp1acbp2* double mutant exhibits EMBL phenotypes and defects in callus induction [38].

Within the PPI network, CBP20 interacts with 64 proteins associated with spliceosome activity, mRNA transport, basal transcription factors, mRNA surveillance and ribosome biogenesis pathways.

In the SE-associated network, 110 proteins were related to spliceosome functions, including nine EMBL-associated genes. Accurate mRNA splicing is essential for embryogenesis because it ensures the generation of mature transcripts required for protein synthesis, developmental regulation, and organogenesis.

Among CBP20-associated proteins, ALY4 (THO complex subunit 4D) functions as an mRNA export adapter involved in the nucleocytoplasmic transport of both spliced and unspliced mRNA.

Efficient mRNA transport mediated by ALY1 proteins is required for normal growth and reproduction. Disruptions affecting ALY4 result in severe vegetative and reproductive defects, including abnormal ovules and reduced seed production.

ALY4 was also associated with SAP18 (histone deacetylase complex subunit SAP18), a component linked to transcriptional repression and chromatin-associated regulation.

SAP18 contributes to tethering the histone deacetylase complex to chromatin and participates in the apoptosis- and splicing-associated protein (ASAP) complex involved in accurate splicing of genes associated with leaf development. *sap18* loss-of-function mutants exhibited a heat-sensitive phenotype, suggesting an additional role in thermoprotection [39].

Consistent with its splicing-related function, SAP18 interacted with 14 proteins associated with spliceosome activity, RNA transport, and mRNA surveillance pathways.

SAP18 was further associated with AGL15 (AGAMOUS-like MADS-box protein 15), a central transcriptional regulator of SE. AGL15 modulates the expression of genes involved in auxin responses and auxin accumulation pathways.

In *A. thaliana,* ectopic expression of *AGL15* directly upregulates *IAA30*, an *AUX/IAA* transcriptional repressor associated with modulation of auxin signaling during SE [40].

Regulatory interactions involving AUX/IAA components and chromatin-associated repressors such as SAP18, HDA19, and TOPLESS (TPL) are discussed in subsequent sections.

*AGL15* also directly regulates AFL (*ABI3/FUS3/LEC2*) genes, which are master regulators of embryogenesis [41]. In addition, *AGL15* promotes embryogenic reprogramming in cooperation with *HDAC6* and *HDAC19*, which influences miR156 abundance during embryogenic induction [42].

Overexpression of *AGL15* promotes SE in *A. thaliana*, soybean, and cotton, whereas *agl15 agl18* double mutants exhibit reduced embryogenic competence [43].

Downstream of *AGL15*, interactions involving FUS3 and LEC2 were identified. Mutations affecting *LEC1* and *FUS3* cause EMBL phenotypes associated with impaired desiccation tolerance during seed maturation [44,45].

*FUS3*, a B3 domain-containing transcription factor, regulates late embryogenesis and contributes to ABA accumulation while negatively regulating gibberellin biosynthesis.

*FUS3* has also been implicated in heat stress-associated developmental regulation by delaying germination under high temperatures.

*LEC2*, another B3 domain-containing transcription factor, is essential for cotyledon identity, suspensor morphology, maturation phase progression, and suppression of premature germination. Ectopic expression of *LEC2* is sufficient to induce SE.

Both LEC2 and FUS3 interacted with LEC1 (NY-YB9), a central NF-Y transcription factor. Together with ABI3, these proteins form the core “LEC module”, a regulatory framework controlling seed maturation and embryogenesis. LEC-1 was associated with NFYA9, NF-YC2, NF-YC3, consistent with the role of NF-Y transcription during embryo development, seed maturation, and SE induction [46]. The SE-associated PPI network contained 16 histone-like CBF/NF-Y transcription factors.

*NF-YA1*, *NF-YA5*, *NF-YA6*, and *NF-YA9* are co-expressed with *LEC1* from the heart to the late torpedo stage of embryogenesis, and their overexpression can induce SE, while *nf-ya1* mutants display embryo defects during the early heart stage.

Overexpression of *LEC1* and *LIL* also induces the expression of *NF-YA1*, *NF-YA5,* and *NF-YA9* [46]. Finally, NF-YB11 interacted with 16 proteins associated with DNA replication and nucleotide excision repair pathways.

These observations suggest that TOR-associated regulatory modules may be functionally connected with RNA processing, chromatin regulation, auxin-responsive transcriptional programs, and the activation of master embryogenic regulators during SE induction.

The enrichment of EMBL-associated genes within these interconnected pathways further supports the importance of coordinated transcriptional and post-transcriptional relations during embryogenic reprogramming.

Importantly, the interaction between TOR-associated modules and LEC/FUS3/AGL15-centered networks positions TOR signaling upstream of core embryogenic transcriptional programs.

This observation supports a model in which TOR signaling may contribute not only to metabolic activation but also to the establishment of embryogenic cell identity.

### 3.4. Interaction of the Master Regulators of SE with Lipid Metabolism

FUS3, LEC2, NFYB9 and DGAT1 interacted with WRI1 (Ethylene-responsive transcription factor WRINKLED1), a master regulator of fatty acid and oil biosynthesis during embryo development.

WRI1 functions as a transcriptional activator involved in the regulation of the sugar-responsive genes and the control of carbon allocation from sucrose metabolism toward lipid accumulation in developing seeds.

WRI1 binds GCC-box promoter elements and contributes to glycolysis, sugar uptake and seed oil accumulation, processes required for embryo development, seed germination and seedling establishment.

Direct interactions between WRI1 and LEC2 enhance the recruitment of *LEC2* to downstream target genes and stimulate their activation. In addition, WRI1 forms regulatory complexes with LEC1, LEC2, and FUS3, promoting auxin and lipid accumulation during somatic embryo induction through activation of *YUCCA4* (*YUC4*) and *OLESIN3* (*OLE3*) [47,48].

**Figure 6:**
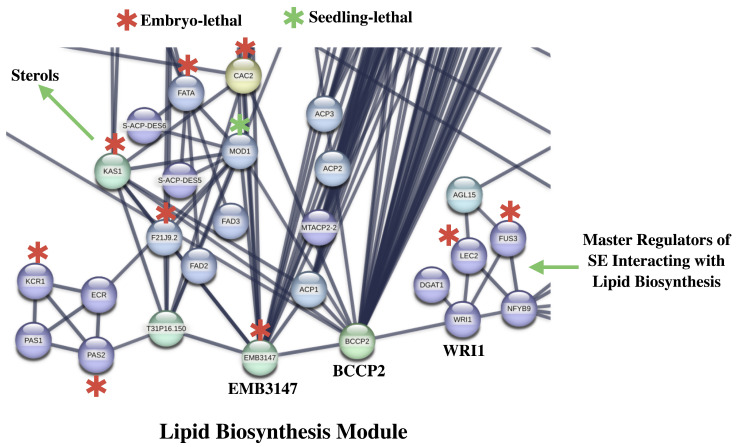
Interactions between the masters of SE regulators and BCCP2-associated lipid biosynthesis pathways in the *A. thaliana* PPI network. The network was generated using STRING v12.0 with the highest confidence score (≥0.900). The green arrows means interacting proteins with the master regulators of SE and sterol proteins.

Within the associated PPI network, WRI1 interacted with five proteins involved in embryo development ending, seed dormancy, SE, and triglyceride and lipid biosynthesis pathways (Figure 6).

WRI1 was also associated with BCCP2 (Biotin carboxyl carrier protein of acetyl-CoA carboxylase 2, chloroplastic), a transcriptional target of WRI1. Elevated expression of BCCP2 contributes to the production of precursors required for fatty acid biosynthesis.

BCCP2 forms part of the acetyl coenzyme A carboxylase (ACCase) complex, in which biotin carboxylase first catalyzes the carboxylation of the carrier protein, followed by transfers of the carboxyl group to generate malonyl-CoA.

ACCase catalyzes the conversion of acetyl-CoA into malonyl-CoA, a key precursor for plastidial fatty acid biosynthesis within multiple cytosolic biosynthetic pathways, including fatty acid elongation.

Mutations affecting *ACC1* (*acc1-1* and *acc1-2*) produce the recessive embryo-lethal (EMBL) phenotype, highlighting the importance of fatty acid biosynthesis during embryogenesis [49].

Within the SE-associated network, BCCP2 interacted with 38 proteins related to oxidative phosphorylation, fatty acid metabolism, carbon metabolism, and propanoate metabolism pathways.

These observations suggest that TOR-associated embryogenesis regulatory networks may be functionally linked to lipid biosynthesis and carbon allocation pathways required for somatic embryo formation.

The integration of WRl1-, LEC-, and ACCase-associated modules further supports the importance of metabolic reprogramming and fatty acid biosynthesis during SE induction.

### 3.5. BCCP2 Interaction with Lipid Biosynthesis

BCCP2 interacted with EMB3147 (MCM7), an acyl-carrier-protein S-malonyltransferase involved in the transfer of malonyl groups from malonyl-CoA to ACP, thereby generating malonyl-ACP required for fatty acid elongation.

EMB3147 localizes to plastids and mitochondria and is highly expressed in proliferating tissues and developing embryos. Null alleles (*mcamt-1* and *mcamt-2*) exhibit EMBL phenotypes characterized by developmental arrest at the globular stage [50]. In contrast, EMB3147 overexpression results in seed yield and storage oil accumulation [50].

Within the SE-associated PPI network, EMB3147 interacted with 17 proteins associated with chloroplast and mitochondrial functions; respirasome activity; fatty acid biosynthesis; metabolism of propanoate, pyruvate, and biotin; and phosphorylation pathways.

BCCP2 and EMB3147 were both connected with KAS1 (3-Oxoacyl-[acyl-carrier-protein] Synthase II), a plastid-localized β-ketoacyl-ACP synthase involved in elongation reactions during de *novo* fatty acid biosynthesis.

KAS1 catalyzes the condensation of malonyl-ACP with acyl-ACP intermediates, driving fatty acid chain elongation beyond C4 [51]. *Kas1* loss-of-function mutants are EMBL [52], whereas partial loss-of-function alleles display reduced chloroplast division, pale-green leaves, and impaired growth consistent with altered membrane lipid biosynthesis [52].

Within the SE-associated network, KAS1 interacted with 10 proteins associated with chloroplast function; fatty acid biosynthesis; and biotin, propanoate and pyruvate metabolism.

BCCP2 and EMB3147 were additionally associated with ACP1 (Acyl carrier protein 1, chloroplastic), a substrate carrier that interacts with multiple enzymes required for fatty acid biosynthesis.

Fatty acids generated by these pathways constitute essential precursors for phospholipid biosynthesis, membrane formation, cellular signaling, and maintenance of membrane fluidity.

EMB3147 was also connected with ACP2 (Acyl carrier protein 2, chloroplastic). Although single *acbp1* and *acbp2* mutants show normal growth, the *acbp1acbp2* double mutant is EMBL, highlighting the importance of lipid-associated pathways during early embryogenesis, a developmental stage characterized by intense membrane biogenesis and cellular proliferation.

Further downstream, EMB3147 interacted with F21J9.2 (3-oxoacyl-[acyl-carrier-protein] reductase, chloroplastic), a member of the short-chain dehydrogenase/reductase (SDR) family involved in chloroplastic fatty acid biosynthesis.

Disruption of F21J9.2 results in EMBL phenotypes, reinforcing the essential role of plastidial fatty acid metabolism during embryo development.

Both BCCP2 and EMB3147 were also associated with MOD1 (Enoyl-[acyl-carrier-protein] reductase I), which catalyzes the final reduction step of the fatty acid elongation cycle, converting trans-2-enoyl-ACP to acyl-ACP using NADH or NADPH [51].

The *mod1* mutant exhibits seedling lethality, dwarfism, chlorosis, defective chloroplast development and severe alteration to fatty acid biosynthesis [51]. Interestingly, the *mod1* mutant also triggers reactive oxygen species (ROS)-mediated runaway cell death, linking fatty acid metabolism with stress and defense signaling pathways [53].

Within the SE-associated network, MOD1 interacted with 10 proteins associated with fatty acid metabolism, unsaturated fatty acid biosynthesis, biotin, and chloroplast/photosynthesis functions.

MOD1 was further associated with FATA (Fatty Acyl-ACP thioesterases), an enzyme involved in hydrolysis of acyl-ACP intermediates to release fatty acids subsequently exported from plastids for membrane and storage lipid biosynthesis [54].

*A. thaliana* encodes two major isoforms, *FATA* and *FATB*, with distinct substrate preferences for unsaturated and saturated acyl-ACP intermediates, respectively. Although *fatb* mutants remain viable, the *fata*/*fatb* double mutant exhibits EMBL phenotypes [55].

Overexpression studies indicate that FATB promotes medium-chain saturated fatty acid accumulation, whereas FATA enhances unsaturated fatty acid biosynthesis and stress tolerance [54,56].

Within the associated network, FATA interacted with seven proteins related to unsaturated fatty acids, fatty acid metabolism, peroxisome pathways, and secondary metabolite biosynthesis.

BCCP2 and EMB3147 were additionally linked to CAC2 (Biotin carboxylase, chloroplastic), another component of the acetyl-CoA carboxylase complex involved in malonyl-CoA production.

*cac2* mutant exhibits severe defects in fatty acid biosynthesis, chloroplast development, plant growth and photosynthesis, ultimately resulting in EMBL phenotypes [57].

Downstream, CAC2 interacts with T31P16.150 ((3R)-hydroxymyristoyl-[acyl carrier protein] dehydratase). This enzyme catalyzes a dehydration step in the fatty acid elongation cycle, converting beta-hydroxyacyl-ACP into trans-2-enoyl-ACP.

T31P16.150 was further associated with PAS2 (Very-long-chain (3R)-3-hydroxyacyl-CoA dehydratase), a component of VLCFA elongation pathways that catalyze dehydration reactions required for very-long-chain fatty acid biosynthesis [58].

*pas2* mutants are EMBL, while partial loss-of-function alleles (*pas2-1*) show abnormal cell division and cytokinin-associated developmental defects [59]. PAS2 localizes to the endoplasmic reticulum and functions as part of a multienzyme elongase complex [60].

PAS2 also interacted with KCR1 (Very-long-chain 3-oxoacyl-CoA reductase), which catalyzes the second step of VLCFA elongation by reducing 3-oxoacyl-CoA intermediates using NADPH [61].

*kcr1* mutants are EMBL [61], whereas elevated KCR activity has been correlated with increased cuticular wax accumulation and altered seed oil composition [58].

Within the SE-associated network, KCR1 interacted with proteins involved in cuticular wax and unsaturated fatty acid biosynthesis pathways.

These observations suggest that TOR-associated lipid metabolic networks may contribute to extensive membrane biogenesis, carbon allocation, and metabolic remodeling required during SE induction.

The enrichment of EMBL-associated genes through plastidial and VLCFA biosynthetic pathways further supports the importance of coordinated fatty acid metabolism during embryogenic reprogramming.

### 3.6. Lipid Biosynthesis Interaction with Sterol Biosynthesis

**Figure 7:**
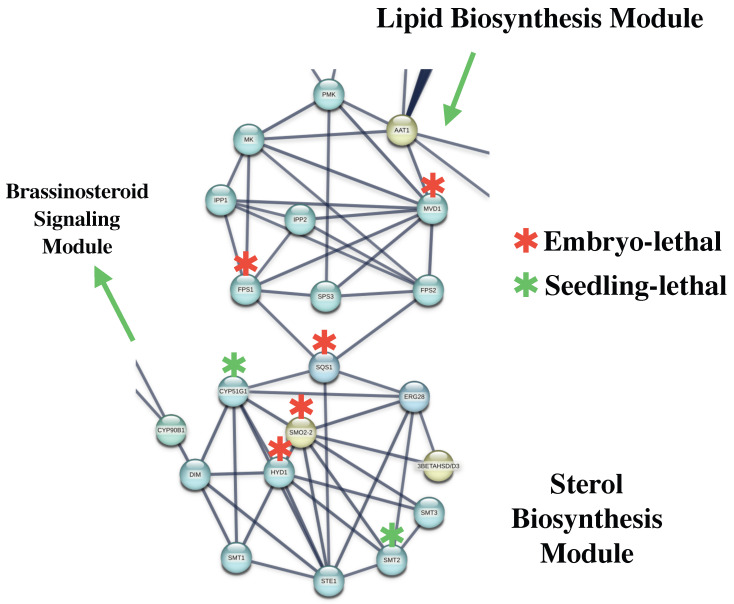
Interaction between lipid biosynthesis pathways and sterol biosynthesis in the *A. thaliana* SE-associated PPI network. The network was generated using STRING v12.0 with the highest confidence score (≥0.900). The green arrows mean the interaction with the lipid biosynthesis module and the brassinosteroid signaling module.

BCCP2 interacted with AAT1 (Acetyl-CoA acetyltransferase 1, cytosolic), an enzyme required for the mevalonate pathway and the biosynthesis of terpenoids and sterols (Figure 7).

Acetyl-CoA acetyltransferases are essential for plant viability because disruption of the mevalonate pathway severely affects sterol biosynthesis, membrane integrity and developmental progression.

In *A. thaliana*, mutations affecting *AACT* genes result in EMBL phenotypes due to impaired terpenoid biosynthesis and altered development regulation.

AAT1 interacted with MVD1 (Diphosphomevalonate decarboxylase1, peroxisomal), an enzyme catalyzing a committed step in the biosynthesis of isoprenoid-derived compounds, including sterols and terpenoids.

MVD1 specifically utilizes (R)-5-diphosphomevalonate (MVAPP) as a substrate. Disruption of MVD1 function reduces campesterol and sitosterol accumulation, leading to developmental abnormalities and reduced seed viability.

Null *mvd1* mutants are EMBL, whereas *mvd2* mutants remain viable but exhibit reduced growth and sterol content [62]. Double knockdowns produce severe sterol deficiency, altered membrane properties, and collapse of root meristems [62].

Within the SE-associated network, MVD1 interacted with eight proteins involved in isoprene, sterol and chlorophyll biosynthesis pathways (Figure 4).

MVD1 was also associated with FPS1 and FPS2 (Farnesyl pyrophosphate synthases), which catalyze sequential condensation reactions generating farnesyl pyrophosphate (FPP), a central precursor for sterol biosynthesis.

*fps1* mutants exhibit seedling lethality and reduced sterol accumulation of upstream intermediates [63]. FPS2 acts partially redundantly with PPS1, and *fps1 fps2* double mutants are EMBL [63].

FPS1 and FPS2 further interacted with SQS1 (Squalene synthase 1), which catalyzes the reductive dimerization of two FPP molecules into squalene, representing the first committed step of sterol biosynthesis.

Loss-of-function mutation of *sqs1* results in dwarfism, male sterility, reduced sterol content, and FPP accumulation [64].

Conversely, overexpression of *SQS1* increases sterol accumulation and biomass production in *A. thaliana* [65].

Sterols participate in multiple developmental and signaling processes, including signal transduction [66], auxin and ethylene signaling, regulation of auxin efflux carrier [67], cellulose biosynthesis [68,69], cell wall development [70], activation of cyclin CycD3 [71], endocytosis, and cytoskeleton organization [72]. Sterols are also essential for embryogenesis [73,74,75], vascular patterning [76], stem elongation [77], plastid development [78], tillering [79], flowering regulation, and potato tuberization [80].

SQS1 was connected with CYP51G (Sterol 14-demethylase), an enzyme catalyzing obtusifoliol 14α-demethylation, an essential step in the production of membrane sterols such as campesterol and sitosterol. In *A. thaliana*, the c*py51A2* mutant exhibits seedling lethality [81].

Overexpression of *CYP51G1* in *Solanum lycopersicum* increases total sterol accumulation without major developmental abnormality [82]. Pharmacological inhibition of *CPY51* using azole fungicides mimics sterol-deficiency phenotypes and disturbs auxin transport pathways [83].

CYP51G1 further interacted with HYD1 (3β-hydroxysteroid-Δ8, Δ7-isomerase), an enzyme involved in the synthesis of 24-methylenelophenol and required for root growth, vascular development [76], developmental patterning [84], and maintenance of polar auxin transport [67].

*hydra1* mutants display EMBL phenotypes characterized by defective apical–basal patterning and altered cell polarity [84].

HYD1 was also associated with SMO2-2, an enzyme required for Δ7-avenasterol biosynthesis and involved in embryonic and post-embryonic development, as well as auxin homeostasis [85].

*smo2-1 smo2-2* double mutants are EMBL, further supporting the role of sterol metabolism in auxin-regulated embryogenesis [85].

In addition, HYD1 interacted with SMT2, which catalyzes the C24-methylation of 24-methylenelophenol to produce sitosterol precursors [86].

In *A. thaliana*, *smt2* mutants show altered sitosterol/campesterol ratios, dwarfism, and reduced fertility [87].

CYP51G1 was also associated with SMT1 (Cycloartenol-C-24-methyltransferase), an enzyme catalyzing the methyl transfer reactions that occur during sterol biosynthesis.

*smt1* mutants display pleiotropic developmental phenotypes including dwarfism, wrinkled leaves, BR deficiency, and defective auxin transport [72]. Moreover, *smt1 smt2* double mutants are embryo-lethal, indicating that disruption of C24-alkylation severely compromises embryogenesis.

These observations suggest that TOR-associated lipid metabolic modules may be functionally linked with sterol biosynthesis pathways required for membrane organization, hormone signaling, and embryogenic development during SE.

The widespread presence of EMBL-associated genes through sterol biosynthesis pathways further supports the importance of coordinated lipid and sterol metabolism during embryogenic reprogramming.

### 3.7. SQS1 Interacting with STE1 and ERG28

SQS1 was further associated with STE1 (Δ (7)-sterol-C5(6)-desaturase 1), an enzyme involved in the biosynthesis of sitosterol and campesterol, important sterol components required for membrane integrity and BR biosynthesis.

*ste1* mutants in *A. thaliana* deficient in C5-SD1 activity accumulate unusual Δ7-sterols and display dwarfism, delayed flowering, and reduced fertility [88].

SQS1 was also connected with ERG28 (Ergosterol biosynthetic protein 28), a component associated with sterol biosynthesis and polar auxin transport.

In *A. thaliana*, *erg28* mutants display phenotypes consistent in altered auxin transport, including pin-shaped inflorescence, reduced apical dominance, organ fusion, and impaired root growth [89].

These observations further support the functional association between sterol biosynthesis pathways and auxin-regulated developmental processes during SE.

The integration of SQS1, STE1, and ERG28 within the SE-associated network suggests that sterol metabolism may contribute not only to membrane biogenesis but also to hormone-dependent developmental patterning embryogenic reprogramming.

Collectively, these interconnected lipid, sterol, and BR-associated pathways suggest the existence of a coordinated metabolic regulatory axis supporting membrane biogenesis, hormone signaling, cellular proliferation, and developmental patterning during embryogenic reprogramming.

The extensive enrichment of EMBL-associated genes across these pathways further reinforces the essential role of lipid metabolic homeostasis during embryogenesis.

### 3.8. Sterol Interaction with BR Biosynthesis

**Figure 8:**
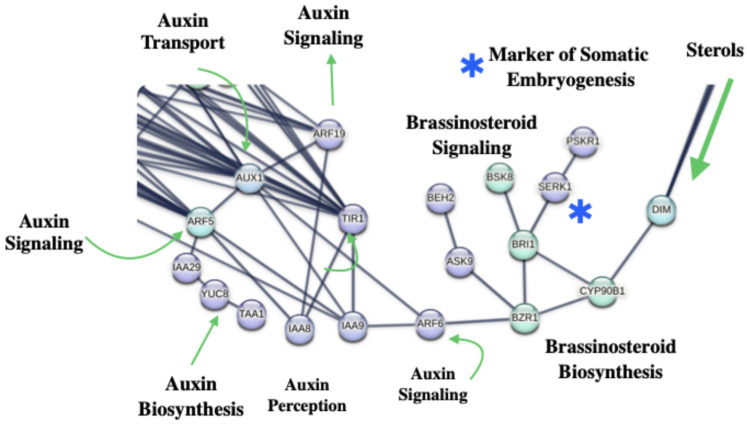
Interaction between sterol metabolism, BR signaling, and auxin signaling pathways in the *A. thaliana* SE-associated PPI network. The network was generated using STRING v12.0 with the highest confidence score (≥0.900). The green arrow means the interaction of the sterol biosynthesis module with the auxin signaling module.

CYP51G1, HYD1, SMT1 and STE1 were associated with DIM (Δ24--sterol reductase), an enzyme required for campesterol biosynthesis, an early precursor of brassinolide (Figure 8).

DIM participates in the conversion of 24-methylenecholesterol into campesterol and is also involved in the conversion of isofucosterol into sitosterol. Through this activity, DIM contributes to sterol homeostasis and BR biosynthesis.

*dim* mutants exhibit severe dwarfism and reduced fertility due to impaired cell elongation and BR deficiency. This phenotype can be rescued by exogenous brassinolide application. In addition, *dim* mutants accumulate 24-methylenecholesterol and exhibit reduced campesterol levels, consistent with disruption of BR precursor biosynthesis [90].

Within the SE-associated PPI network (Figure 7), DIM interacted with CYP90B1 (DWF4; Cytochrome P450 90B1), an enzyme that catalyzes the C22 α-hydroxylation step during BR biosynthesis.

DWF4 converts campestanol to 6-deoxocathasterone and 6-oxocampestanol into cathasterone.

*dwf4* mutants display severe dwarf phenotypes associated with impaired cell elongation and can also be rescued by BR application.

DWF4 was further associated with BZR1 (BRASSINAZOLE-RESISTANT 1), a transcription factor regulating BR-responsive gene expression. BZR1 binds the BR response element (BRRE) within the target promoter and functions as a major downstream regulator of BR signaling.

BZR1 contributes to developmental regulation by modulating genes involved in ovule development, including ANT and AP2, and participates in feedback regulation of BR biosynthesis.

BZR1 interacted with BRI1 (BRASSINOSTEROID INSENSITIVE 1), a dual-specificity receptor kinase that phosphorylates serine/threonine- and tyrosine-containing substrates.

Upon BR binding, *BRI1* activates signaling cascades involved in cell elongation, flowering, chloroplast senescence, stress-response gene expression, and overall plant development.

BRI1 binds brassinolide and castasterone and may additionally participate in feedback regulation of BR biosynthesis.

BRI1 further interacted with SERK1 (Somatic Embryogenesis Receptor Kinase 1), a receptor-like kinase associated with embryogenic competence and BR signaling.

SERK1 phosphorylates BRI1 and CDC48 and acts redundantly with SERK2 during sporophytic development and male gametophyte formation.

In addition to its developmental roles, SERK1 has widely been associated with the acquisition of embryogenic competence during SE induction.

SERK1 was also associated with PSKR1 (Phytosulfokine receptor 1), a receptor kinase possessing both serine/threonine kinase and guanylate cyclase activity.

Upon phytosulfokine binding, PSKR1 regulates signaling pathways involved in cell differentiation, organogenesis, SE, cellular proliferation, and plant growth.

PSKR1 additionally contributes to immune response and forms signaling complexes involving BAK1 and other receptor-like kinases. In this pathway, CNGC17 and plasma membrane H+-ATPase (AHAs) function as downstream ion transport components activated by PSKR1/BAK1-associated complexes.

Notably, *serk1 serk2* double mutants fail to develop a functional tapetal layer and exhibit male sterility [91].

These observations suggest that TOR-associated sterol metabolic pathways may be functionally connected with BR signaling networks involved in cellular proliferation, embryogenic competence, and developmental reprogramming during SE.

The integration of sterol biosynthesis enzymes, BR receptors, and SERK-associated signaling modules further supports the importance of hormone-mediated developmental regulation during somatic embryogenesis.

### 3.9. BRs Interacting with Auxin Signaling

Oxidative modification enhances *BZR1* transcriptional activity by promoting its interaction with regulators involved in auxin and light-signaling pathways, including AUXIN RESPONSE FACTOR6 (ARF6) [92].

Auxin response factors (ARFs) are transcription factors that bind auxin-responsive promoter elements (AuxREs) containing the consensus sequence 5′-TGTCTC-3′.

ARF proteins regulate the transcription of auxin-responsive genes, and their activity is modulated through interaction with Aux/IAA transcriptional repressors.

ARF6 functions primarily as a transcriptional activator involved in reproductive development, including stamen and gynoecium maturation, and contributes to jasmonic acid biosynthesis. ARF6 acts partially redundantly with ARF8.

Whitin the SE-associated PPI network, auxin signaling-related pathways comprise 114 proteins associated with Aux/IAA protein, auxin biosynthesis, auxin perception, ubiquitin-mediated proteolysis, mitotic cell cycle regulation, mRNA surveillance, serine/threonine phosphatase complexes, protein processing in the endoplasmic reticulum, and SIN3-associated chromatin regulation pathways.

ARF6 interacted with AUX1, a protein-driven auxin influx carrier involved in establishing auxin gradients from developing leaves toward root tips by facilitating auxin loading into vascular tissues (Figure 8).

AUX1 was also connected with ARF5 (MONOPTEROS), a central auxin response factor regulating embryo axis formation and vascular tissue differentiation.

ARF binds AuxRE-containing promoters and functions primarily as a transcriptional activator. Interactions with Aux/IAA proteins modulate their activity during early auxin response.

ARF5 additionally acts redundantly with ARF7 and counterbalances AMP1-associated developmental pathways.

Within the SE-associated network, ARF5 interacted with 16 proteins involved in auxin signaling, transcriptional regulation, and embryo/post-embryonic development. ARF5 further interacted with IAA8, a member of the Aux/IAA family of short-lived transcriptional repressors. Aux/IAA proteins repress auxin-response gene expression at certain auxin concentrations through interaction with ARFs, whereas auxin-induced degradation of Aux/IAAs relieves this repression.

IAA8 was further associated with ARF19, another auxin response factor involved in lateral root formation and ethylene-responsive pathways. SRF119 directly regulates LBD16 and LBD29 and acts partially redundantly with ARF7. Similarly to other ARFs, ARF19 is modulated through interactions with Aux/IAA proteins.

ARF19 was also connected with AUX1, which mediates auxin influx and contributes to acropetal and basipetal auxin transport within the root apex. AUX1 facilitates auxin unloading from mature phloem tissues into the root meristem and contributes to the establishment of an auxin gradient for developmental patterns.

In addition, AUX1 interacts with AXR1 (NEDD8-activating enzyme E1 regulatory subunit AXR1), a component of the RUB1/NEDD8 conjugation pathways required for auxin signaling.

AXR1 participates in activation of RUB1/NEDD8 proteins through adenylation and thioester formation reactions that ultimately regulate cullin-RING ubiquitin ligase complexes.

AXR1 also contributes to meiotic recombination and chromosome synapsis through activation of CRL4-associated pathways.

AXR1 interacted with RUB1, a ubiquitin-like modifier required for embryogenesis. Simultaneous disruption of *RUB1* and *RUB2* causes developmental arrest at the two-cell stage of embryogenesis, highlighting the essential role of RUB conjugation during plant development [93,94].

RNAi-mediated suppression of RUB proteins additionally produces severe post-embryonic developmental abnormalities and altered jasmonic acid and ethylene signaling pathways.

Mutations affecting RUB conjugation components such as AXR1 and RCE1 stabilize AUX/IAA repressors and reduce auxin responsiveness.

RUB1 further interacted with RBX1A, a RING-H2 finger protein functioning as a core subunit of SCF E3 ubiquitin ligases complexes. RBX1 recruits the ubiquitin-conjugating E2 enzyme and contributes to RUB-mediated modification of CUL1, an essential component of SCF ubiquitin ligases. Elevated RBX1 expression increases RUB-modified CUL1 accumulation and stabilizes SCF substrates such as AXR2/IAA7, thereby altering auxin signaling responses [95].

RBX1A was additionally associated with CSN4, a subunit of the COP9 signalosome complex involved in photomorphogenesis, auxin signaling and jasmonate-responsive pathways. The COP9 signalosome regulates culling-RING ligase activity through deneddylation of cullin subunits, thereby modulating ubiquitin-mediated protein degradation pathways.

CSN4 further interacted with RBX1A and SKP1A, an adaptor component of SCF E3 ubiquitin ligase complexes. Together with CUL1, RBX1 and F-box protein, SKP1A contributes to the assembly of SCF complexes with distinct developmental functions depending on the associated F-box protein.

These include SCF(TIR1) in auxin signaling, SCF(COl1) in jasmonate signaling, SCF(EID1) and SCF(AFR) in phytochrome A signaling, SCF(ORE9) in senescence, and SCF(EBF1/EBF2) in ethylene signaling pathways. SKP1A also contributes to embryogenesis, post-embryonic development, chromosome segregation, and cellular proliferation.

These observations suggest that TOR-associated BR and auxin signaling networks may converge through ubiquitin-mediated proteolysis and SCF-dependent regulatory pathways during SE induction.

The integration of ARF-, AUX/IAA-, RUB-, COP9-associated modules further supports the importance of hormone-responsive transcriptional regulation and controlled protein turnover during embryogenesis reprogramming.

These observations support the existence of extensive crosstalk between TOR signaling, BR-mediated developmental regulation, auxin transport, and ubiquitin-dependent proteolysis during SE induction; such integration may enable rapid and dynamic remodeling of transcriptional responses required for embryogenic fate acquisition.

### 3.10. TOR Signaling Interacting with Ribosomal Proteins

**Figure 9:**
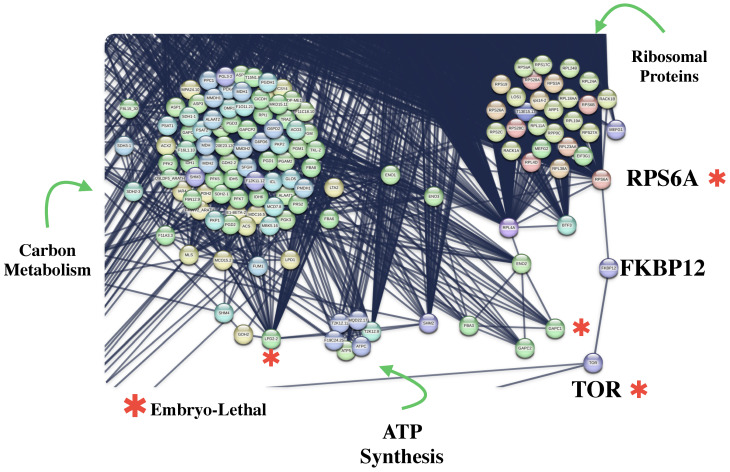
Interaction between TOR signaling and ribosome-associated protein in the *A. thaliana* SE-associated PI network. In the PPI network of SE in *A. thaliana*, TOR interacts with RPS6A. The network was generated using STRING v12.0 with the highest confidence score (≥0.900). The green arrows mean the carbon metabolism and ATP synthesis module interacting within the network.

Within the SE-associated PPI network (Figure 9), 32 ribosomal proteins interacted with the TOR module, highlighting the extensive association between TOR signaling and translational regulation during somatic embryogenesis (Figure 9).

TOR-mediated regulation of rRNA synthesis and protein production is essential for developmental processes such as embryogenesis, flowering, growth and senescence.

Mutations affecting TOR signaling or RPS6A delay plant growth and development progression, demonstrating the importance of this pathway in coordinating cellular growth response [96].

TOR interacted with FKBP2 (Peptidyl-prolyl cis-trans isomerase FKBP12), a peptidyl isomerase (PPIases) involved in protein folding through catalysis of cis-trans isomerization of proline peptide bonds. FKBP2 is also known to mediate rapamycin-dependent inhibition of TOR kinase activity.

FKBP12 further interacts with RPS6A (Ribosomal protein S6A), a ribosomal protein involved in translational regulation and cellular proliferation. RPS6A may contribute to selective translation of specific classes of mRNA associated with growth and developmental regulation [96].

In the PPI network of SE, RPS6A interacted with 20 ribosomal proteins and five proteins associated with ribosome biogenesis pathways linked to embryogenic development.

In addition, LEC2 was associated with RPL14B, further connecting embryogenic transcriptional regulators with ribosome-associated translational machinery.

These observations suggest that TOR-associated translational modules may contribute to increased ribosome biogenesis and selective protein synthesis required during SE induction.

### 3.11. TOR Interacting with TAP46: One-Carbon Metabolism and Cell Cycle and DNA Repair

The integration of DNA replication, nucleotide biosynthesis, and repair-associated pathways suggests that maintenance of genome integrity may constitute a central requirement during somatic-to-embryogenic cell fate transition.

**Figure 10:**
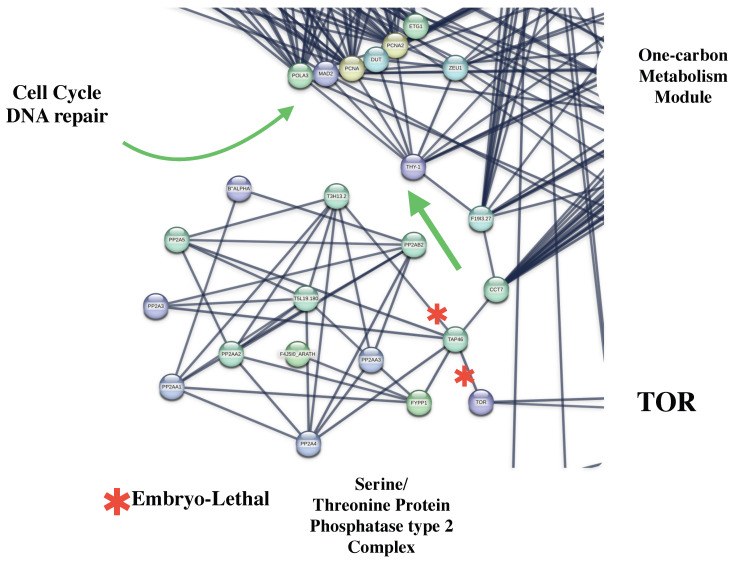
TOR-TAP46-associated interaction with one-carbon metabolism, cell cycle regulation, and DNA repair pathways in the *A. thaliana* SE-associated PPI network. Green arrow associated with TOR explains the interaction with the Serine/Threonine Protein Phosphatase type 2 complex. The arrow close to THY1 means the interaction with one-carbon metabolism and cell cycle repair module.

In the SE-PPI network (Figure 10), TOR interacted with TAP46 (Type 2A phosphatase-associated protein of 46 kDa), a regulatory protein involved in Ser/Thr phosphatase-mediated control of transcription, translation, and cell cycle regulation [97].

TAP46 interacted with seven proteins involved in mitotic cell cycle progression and G1/S transition pathways.

TAP46 was connected with FYPP1 (Phytochrome-associated serine/threonine-protein phosphatase 1), a phosphatase involved in dephosphorylation of phytochromes, particularly their Pfr active forms. PYPP1 contributes to photoperiodic regulation of flowering time through modulation of phytochrome signaling pathways.

TAP46 also interacted with PP2A4 (Serine/threonine-protein phosphatase PP2A catalytic subunit 4), a phosphatase functioning redundantly with PP2A3 during embryogenesis.

PP2A4 contributes to the establishment of auxin gradients, apical–basal polarity and maintenance of root and shoot apical meristems. PP2A4 may additionally regulate polar auxin transport through dephosphorylation of PIN1 and modulation of its subcellular localization.

Furthermore, holoenzyme complexes containing PP2AA1, PP2A4 and B’ZETA or B’ETA subunits negatively regulate innate immunity signaling through modulation of the BAKK1 phosphorylation state in plasma membrane receptor complexes.

TAP46 interacts with CCT7 (T-complex protein 1 subunit eta), a molecular chaperone belonging to the TCP-1 chaperonin family. CCT7 assists protein folding through ATP-dependent mechanisms and has been implicated in the folding of cytoskeletal proteins such as actin and tubulin.

CCT7 interacts with F19I3.27 (phosphoribosylaminoimidazolecarboxamide formyltransferase), an enzyme associated with one-carbon metabolism and nucleotide biosynthesis pathways.

An enzyme, F19I3.27, was further connected with THY-1 (Bifunctional dihydrofolate reductase-thymidylate synthase 1), a bifunctional enzyme involved in folate metabolism and de novo dTMP biosynthesis.

THY-1 catalyzes reactions required for tetrahydrofolate recycling, glycine biosynthesis, purine biosynthesis, and conversion of dUMP to dTMP, thereby contributing to DNA precursor synthesis and cellular proliferation (Figure 10).

THY-1 interacted with PCNA2 (Proliferating cell nuclear antigen 2), an auxiliary factor of DNA polymerase delta involving DNA replication and genome maintenance.

PCNA2 enhanced polymerase processibility during DNA elongation and has been implicated in U resistance and DNA-associated pathways. Although single *dhfr1* and *dhfr2* mutants do not display a visible developmental phenotype, *dhfr-ts1 dhfr-ts2* double mutants are EMBL [98].

In the PPI network, THY-1interacts with 21 different proteins involved in cell cycle regulation, DNA repair, mitosis, ribosomal protein modules, and protein SUMOylating processes (Figure 10).

**Figure 11:**
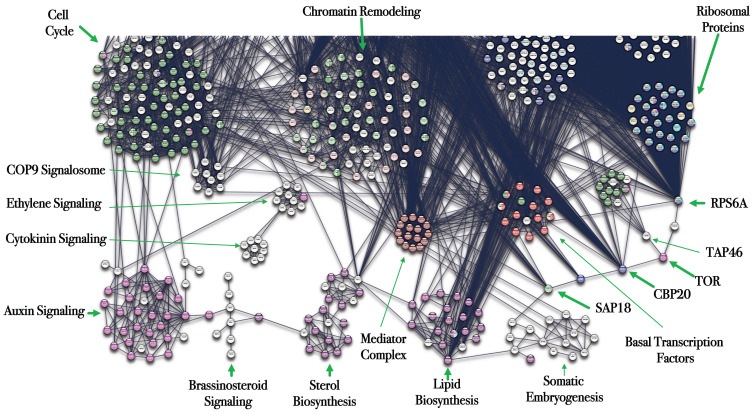
TOR-centered protein interaction network associated with somatic embryogenesis. Direct TOR interactors are distinguished from secondary and higher-order connections identified through STRING topology (confidence ≥ 0.900). Several embryogenesis regulators are linked to TOR through intermediary proteins, including CBP20, and therefore represent indirect functional associations rather than demonstrated direct TOR interactions. The network highlights biologically connected pathways potentially associated with embryogenic reprogramming and provides a framework for future experimental validation.

The TOR-associated modules described in Figure 11 should be interpreted as interconnected regulatory landscapes rather than as evidence of direct TOR-mediated regulation of all associated components. To address this limitation, we distinguished direct protein interactions involving TOR from indirect functional associations inferred through network topology and intermediary nodes. Although several embryogenesis-related regulators are connected to TOR through proteins such as CBP20 and other highly connected hubs, previous experimental studies support the functional relevance of these pathways within TOR-dependent developmental programs.

Therefore, the associations identified in Figure 11 provide a systems-level framework for understanding how TOR signaling may be integrated with transcriptional regulation, RNA processing, proteostasis, metabolism, and embryogenic competence. However, the present network analysis does not establish direct regulatory relationships, and the proposed connections should be considered hypothesis-generating predictions that require experimental validation.

### 3.12. Embryo-Lethal (EMBL) Genes

EMBL genes correspond to essential loci whose loss of function leads to embryo arrest or developmental failure. These genes regulate critical biological processes, including polarity establishment, cell division, genome maintenance, metabolic homeostasis, and organogenesis, during early embryonic development [98].

**Figure 12:**
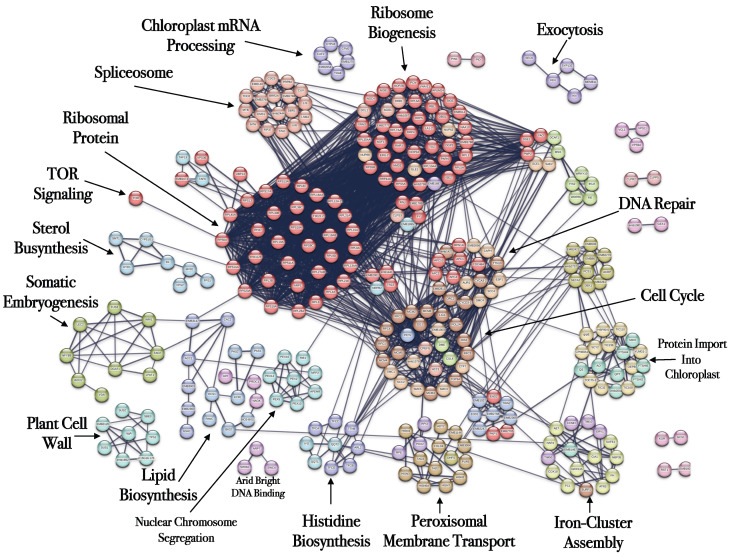
PPI network of EMBL genes from the SE network in *A. thaliana*. STRING v12.0, confidence ≥ 0.900.

Out of 1927 genes analyzed in the SE PPI network, 411 were associated with EMBL phenotypes, highlighting the extensive enrichment of essential developmental regulators throughout the network (Figure 12).

**Figure 13:**
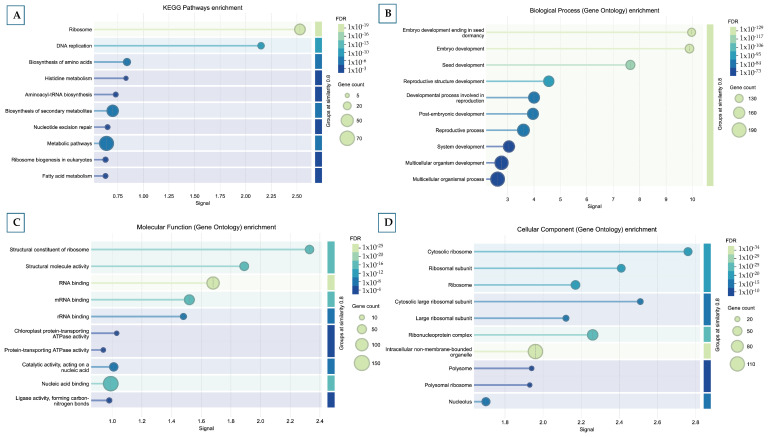
EMBL gene enrichment analyses of GO biological, cellular, molecular and KEGG pathway enrichment.

EMBL-associated genes were distributed across multiple functional modules, including transcriptional regulation, ribosome biogenesis, lipid and sterol metabolism, hormone signaling, DNA repair, ubiquitin-mediated proteolysis, and cell cycle regulation (Figure 13). The complete list of EMBL-associated genes identified in the SE-associated network is provided in Supplementary Table S3.

TOR-TAP46-associated modules may therefore coordinate proliferative activation while simultaneously preventing replication-associated genomic instability.

### 3.13. Model: TOR Signaling Activates SE via Multi-Layered Pathways

**Figure 14:**
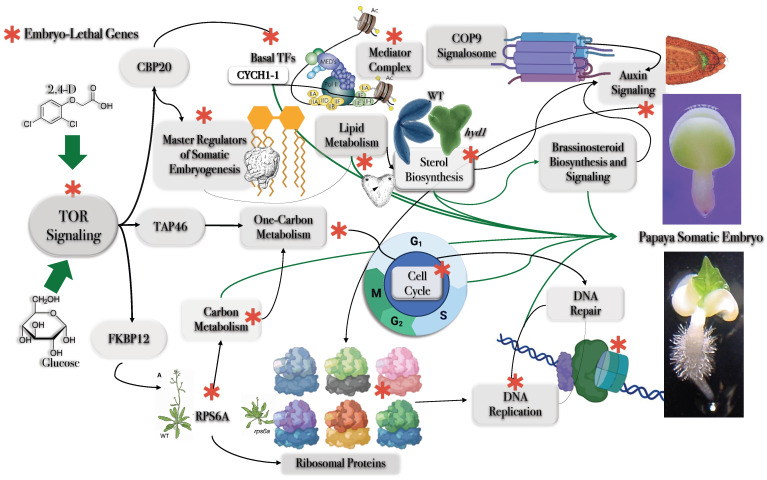
Proposed model of SE molecular mechanisms driven by TOR under 2,4-D treatment (2 mg/L) in *A. thaliana*. TOR signaling relates to transcriptional regulation, translational control, metabolic re-programming, hormone signaling, and genome maintenance pathways through the CBP20, FKBP12, and TAP46 regulatory axes. The model was constructed based on high-confidence inter-actions identified in the SE-associated PPI network generated using STRING v12.0 (confidence score ≥ 0.900). Red asterisks indicate biological processes containing embryo-lethal (EMBL) genes.

The integrated model proposed in Figure 14 illustrates how TOR signaling may contribute to SE via three interconnected regulatory axes:

(i) The TOR–CBP20 axis, associated with transcriptional reprogramming through interactions involving basal transcription factors, the Mediator complex, RNA polymerase-associated pathways, and SE master regulators. This regulatory branch is additionally connected with lipid, sterol, BR, and auxin biosynthesis pathways.
(ii) The TOR–FKBP12 axis, associated with translation regulation through RPS6A activation, ribosome biogenesis and connection with central carbon metabolism pathways.
(iii) The TOR–TAP46 axis, linked with one-carbon metabolism, nucleotide biosynthesis, cell cycle progression, and DNA repair.

These three axes converge on molecular pathways associated with cellular proliferation, developmental reprogramming, metabolic coordination, and genome stability during SE induction.

Traditionally, TOR signaling has been viewed primarily as a cytoplasmic pathway controlling translation, ribosome biogenesis, and cellular growth.

However, accumulating evidence indicates that TOR also exerts nuclear functions that directly influence transcriptional regulation. In *A. thaliana*, the TOR–EIN2 signaling axis provides a mechanistic link between TOR activity and nuclear gene regulation, where TOR-mediated phosphorylation controls the nuclear localization of EIN2 and thereby modulates large-scale transcriptional reprogramming associated with growth and developmental processes.

Notably, disruption of this pathway compromises the expression of numerous TOR-responsive genes involved in DNA replication, cell wall biosynthesis, lipid metabolism, and other developmental functions [99].

Furthermore, recent studies in animal systems have demonstrated that nuclear mTORC1 constitutes a functionally distinct signaling pool capable of directly regulating transcription through chromatin-associated mechanisms, providing evidence that TOR signaling can operate within the nucleus independently of its canonical lysosomal functions [100]. The model was construed based on high-confidence interactions identified in the SE-associated PPI network generated using STRING v.12.0 (confidence ≥ 0.900) (Figure 14).

Together, these findings support the biological plausibility of the transcriptional and chromatin-associated modules identified in our network and suggest that TOR-mediated regulation during SE may involve both indirect cytoplasmic signaling cascades that converge on nuclear regulators and more direct nuclear functions associated with transcriptional control.

Nevertheless, the present network analysis does not establish the subcellular localization of the identified interactions; therefore, these associations should be interpreted as hypothesis-generating rather than direct evidence for nuclear TOR-containing regulatory complexes.

## 4. Conclusions

In summary, this systems-level analysis identifies TOR signaling as a potential master integrator of embryogenic reprogramming during 2,4-D-induced somatic embryogenesis in *A. thaliana*.

The reconstructed interactome reveals extensive coordination among transcriptional regulators, ribosome biogenesis, lipid and sterol metabolism, hormone signaling pathways, ubiquitin-mediated proteostasis, and genome-maintenance mechanisms.

The enrichment of EMBL genes across these modules further emphasizes the developmental relevance of the identified network architecture.

Collectively, these findings provide a conceptual framework for understanding the regulatory architecture underlying somatic embryogenesis in *A. thaliana* and identify candidate pathways for future functional studies. The extent to which these regulatory relationships are conserved in other plant species remains to be experimentally determined.

Collectively, these findings support a potential role for TOR as an integrative signaling hub coordinating developmental, metabolic, and transcriptional programs during somatic embryogenesis. However, the proposed framework remains a hypothesis-generating model, and future experimental studies will be required to validate the predicted TOR-dependent regulatory mechanisms.

### Future Directions

The molecular mechanisms discussed here represent only a partial overview of the molecular events involved in SE development. Additional genes and regulatory pathways certainly may contribute to the establishment and development of SE.

We have applied successfully the rationale described in Section 3.3, Section 3.4 and Section 3.5 (TOR-CBP20—master regulators of SE lipid biosynthesis) to activate SE induction, maturation and plant conversion of common bean *Phaseolus vulgaris*.

## Data Availability

No new data were created or analyzed in this study. Data sharing is not applicable to this article.

## Supplementary Materials

The following supporting information can be downloaded at https://www.mdpi.com/article/10.3390/ijms27146191/s1.

## Abbreviations

The following abbreviations are used in this manuscript:

2,4-D	2,4-dichlorophenoxy acetic acid
TOR	Target of Rapamycin
SE	Somatic Embryogenesis
EMBL	Embryo-Lethal
PPI	Protein–Protein Interaction
BRs	Brassinosteroids
DEG	Differentially Expressed Gene
