# Supplementary material for: TOR Signaling as a Central Integrator of Embryogenic Reprogramming During 2,4-D-Induced Somatic Embryogenesis
Source: Int J Mol Sci. 2026 Jul 10;27(14):6191. doi: 10.3390/ijms27146191 (PMC13409849; doi:10.3390/ijms27146191)
Supplement: Supplementary file 1 [file ijms-27-06191-s001.zip › Supplementary Figure S1.pdf]

International Journal of
Molecular Sciences

MDPI

Review

TOR Signaling as a Central Integrator of Embryogenic Reprogramming during 2,4-D-Induced Somatic Embryogenesis.

José Luis Cabrera-Ponce [1,*], Alex Ricardo Bermudez-Valle [2], Maria del Rosario Cárdenas-Aquino [3], Andrea Maria Navarro-Vega [4], Braulio Uribe-Lopez [5], Aaron Barraza-Celis [6], Eliana Valencia-Lozano [7,*], and Lisset [1] Departamento de Ingeniería Genética, PlanTECC, Departamento de Ingeniería Genética, Centro de Investigación y de Estudios Avanzados del IPN, Unidad Irapuato, Irapuato, Guanajuato, México. 36824, jlcabre@yahoo.com.mx (J. L, C.-P.).

Supplementary material

RPS6A

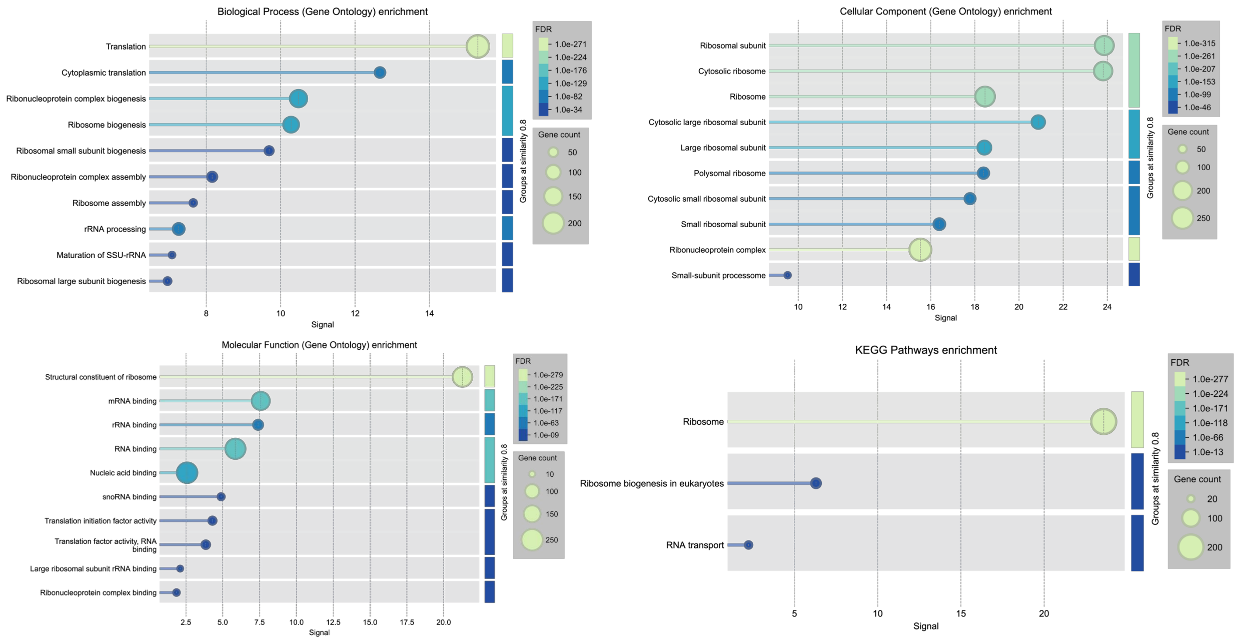

International Journal of
Molecular Sciences

MDPI

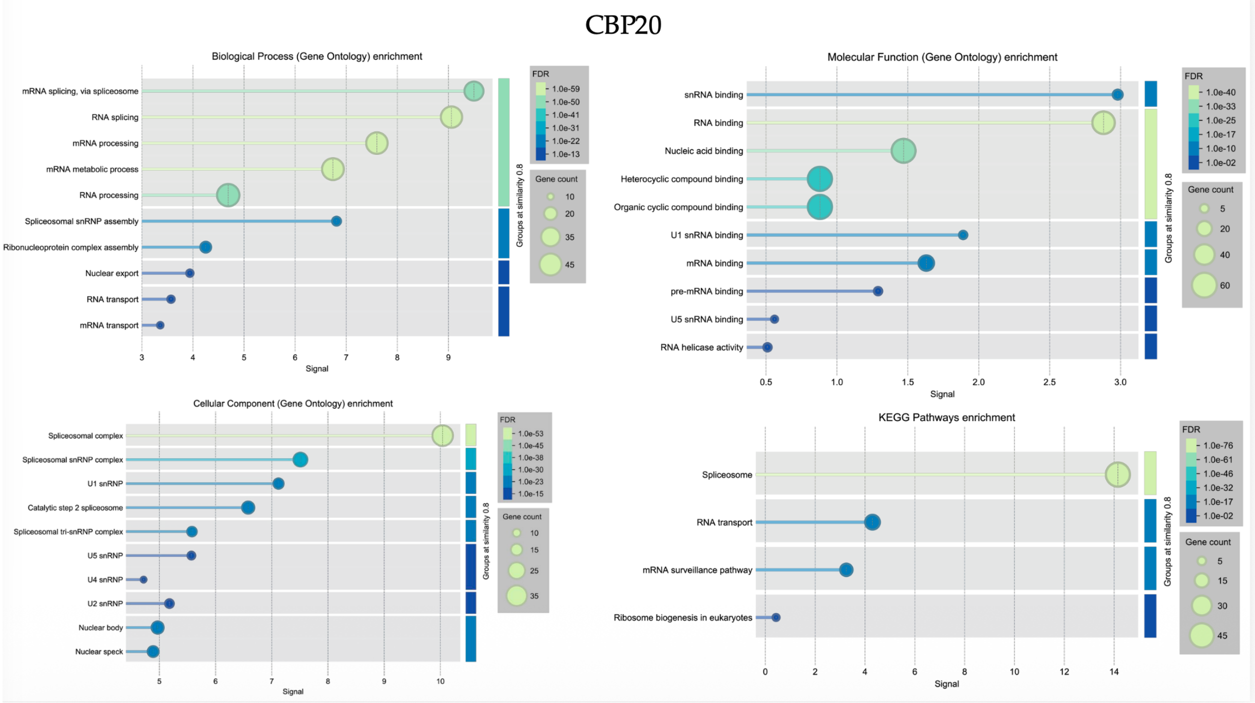

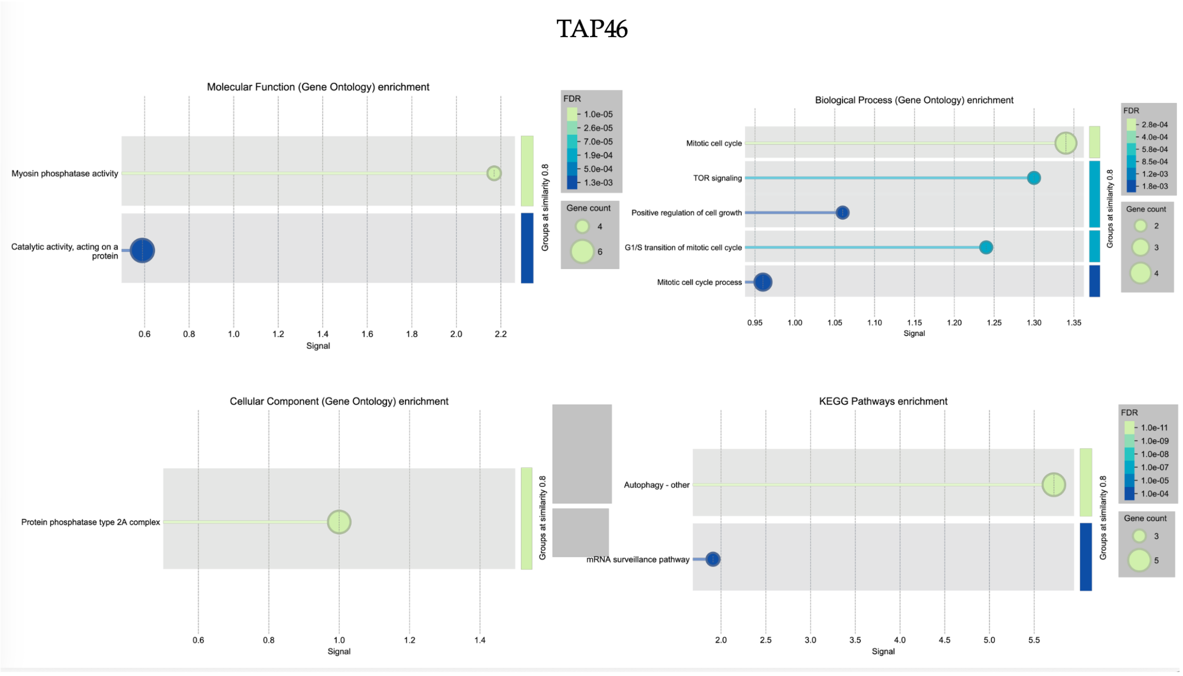

Supplementary Figure S1. Functional enrichment analysis of TOR-Associated Regulatory Axes.
