# Supplementary material for: TOR Signaling as a Central Integrator of Embryogenic Reprogramming During 2,4-D-Induced Somatic Embryogenesis
Source: Int J Mol Sci. 2026 Jul 10;27(14):6191. doi: 10.3390/ijms27146191 (PMC13409849; doi:10.3390/ijms27146191)
Supplement: Supplementary file 1 [file ijms-27-06191-s001.zip › Supplementary Figure S2.pdf]

International Journal of *Molecular Sciences*

Review

TOR Signaling as a Central Integrator of Embryogenic Reprogramming during 2,4-D-Induced Somatic Embryogenesis.

José Luis Cabrera-Ponce [1,*], Alex Ricardo Bermudez-Valle [2], Maria del Rosario Cárdenas-Aquino [3], Andrea Maria Navarro-Vega [4], Braulio Uribe-Lopez [5], Aaron Barraza-Celis [6], Eliana Valencia-Lozano [7,*], and Lisset [1] Departamento de Ingeniería Genética, PlanTECC, Departamento de Ingeniería Genética, Centro de Investigación y de Estudios Avanzados del IPN, Unidad Irapuato, Irapuato, Guanajuato, México. 36824, jlcabre@yahoo.com.mx (J. L, C.-P.).

Supplementary material

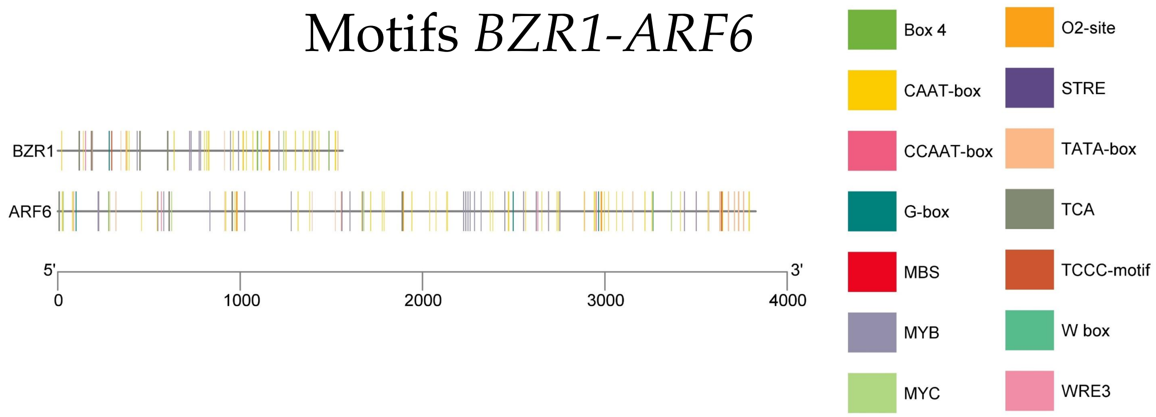

Supplementary Figure S2. Promoter cis-element analysis of ARF6 and BZR1 based on PlantCARE prediction. Distribution of predicted cis-regulatory elements within the 4-kb promoter regions of ARF6 and BZR1. Different types of hormone-responsive, developmental, and stress-related motifs, including G-box, MYB, MYC, W-box, STRE, MBS, and TCA-related elements, are indicated. The identified cis-element profiles reveal shared regulatory features between both promoters, suggesting potential transcriptional convergence associated with brassinosteroid and auxin signaling pathways during somatic embryogenesis.
